# Passive atomic-scale optical sensors for mapping light flux in ultra-small cavities

**DOI:** 10.1038/s41598-023-32010-y

**Published:** 2023-03-31

**Authors:** Pavao Andričević, Elaine L. Sellwood, Martha-Cary Eppes, Myungho Kook, Mayank Jain

**Affiliations:** 1 Department of Physics, Technical University of Denmark, DTU Risø Campus, 4000 Roskilde, Denmark; 2 Department of Geography & Earth Sciences, University of North Carolina at Charlotte, Charlotte, NC USA

**Keywords:** Physics, Optical physics

## Abstract

Understanding light propagation and attenuation in cavities is limited by lack of applicable light sensing technologies. Here we demonstrate the use of light-sensitive metastable states in wide bandgap aluminosilicates (feldspar) as passive optical sensors for high-resolution mapping of light flux. We develop non-destructive, infrared photoluminescence (IRPL) imaging of trapped electrons in cracks as thin as 50 µm width to determine the spatio-temporal evolution of light sensitive metastable states in response to light exposure. Modelling of these data yields estimates of relative light flux at different depths along the crack surfaces. Contrary to expectation, the measured light flux does not scale with the crack width, and it is independent of crack orientation suggesting the dominance of diffused light propagation within the cracks. This work paves way for understanding of how light attenuates in the minutest of cavities for applications in areas as diverse as geomorphology, biology/ecology and civil engineering.

## Introduction

Our ability to measure light flux in small cavities is limited by the physical size of light sensors. Yet there are many potential applications, especially in natural systems, which would benefit from knowledge of average light flux retrospectively over extended periods of time, e.g. historical or even pre-historic time scales. For instance, in order to document how microorganisms respond to different daylight exposure conditions in small/narrow cavities, or even on different surfaces, we need to be able to measure light fluxes with high spatial resolution on the relevant interfaces^1^. Similarly, rates of photochemical reactions such as photocatalysis^2^ or photosynthesis^3,4^ are all related to light flux, which can only be measured today using coarse active sensors. Lastly, for example, moisture content at crack tips in both natural and man-made materials hastens the material cracking through its influence both on water-dependent stress loading processes like those related to freezing as well as on the subcritical crack (SCC) bond-breaking processes themselves^5–8^. Daylight exposure has been shown to reduce this moisture content, and thus is conceived to have a mitigating impact on crack propagation^9,10^. However, there are no methods today for actually measuring the light flux at crack tips in order to validate these theoretical models and laboratory experiments in natural settings.

To fully understand the role of light in all of these systems and many other, it is critical that we can determine light flux over spatial scales over which the physical and biological processes are operating. Equally, it is important that the detection system itself does not alter the physical process by obstructing the light flux. Ideally, especially for the environmental processes, we want to develop a light sensing technology, based on submicron sized sensors, that are widely available and can provide light fluxes retrospectively (passive readout) in order to understand light induced processes that occur over historical or even prehistorical time scales. Currently, such ideal detectors are not available. Photodiodes (e.g., 0.67 × 0.3 × 0.28 mm)^11^ or light optical fibers (e.g., 100 µm diameter) can be used as active optical sensors in sub-mm sized cavities. However, despite their small sizes such detectors obstruct the light pathways, and the measurements are sensitive to the light collection angle; they are therefore not ideal for high-resolution mapping of light flux. Furthermore, the efficiency of light coupling will vary significantly depending on the scattering geometry; this makes fiber-based detectors non-ideal for measurements on rough surfaces.

In this study, we establish the use of atomic scale, optically sensitive metastable states in the natural aluminosilicate feldspar (K-NaAlSi_3_O_8_), which is one of the most abundant minerals in the Earth’s crust^12^. These metastable states form by the creation of free electrons and holes in the crystal lattice due to its exposure to ionizing radiation from the immediate environment, and subsequent trapping of these free charges in the crystal defects (traps). Upon light exposure, electrons can be released from the trap (detrapping) and eventually annihilate with trapped holes (recombination) to produce luminescence; this process is known as optically stimulated luminescence (OSL)^13^. OSL is widely used to determine absorbed dose (unit Gy = J/Kg) from the ambient ionizing radiation in solid-state dosimetry^14^ and geochronology^15–18^. Particularly in feldspar^15^, the main electron trapping center is known as the principle trap, and its ground state exists at about > 2 eV below the conduction band edge, whereas the first excited state exists at about 1.45 eV above the ground state. The principle trap can be emptied efficiently with light of energy > 1.4 eV (UV to NIR)^19^.

The rate of detrapping or the destruction of the metastable states is a function of light fluence, thus the net depletion of trapped charges depends on both the light flux and the total light exposure time. In a first-order formulation, decay of latent luminescence ($L$) due to light exposure can be described as an exponential function^16^:

1 $$L (t)={L}_{0}{e}^{-\overline{\sigma {\varphi }_{0}}t}$$

where ${L}_{0}$ is the initial luminescence intensity corresponding to the number of occupied traps prior to any light exposure, $t$ (s) is the exposure time, $\sigma$ (cm^2^) is the photoionisation cross section of the electron trap and ${\varphi }_{0}$ (cm^−2^ s^−1^) is the photon flux. $\overline{\sigma {\varphi }_{0}}$ (s^−1^) is the integral of the product $\sigma \times {\varphi }_{0}$ over the relevant bleaching wavelength range, and it represents the effective decay constant of the latent luminescence at the rock surface; thus, by measuring luminescence depletion with exposure time, one can derive the decay constant. Furthermore, Eq. 1 can be used to make a relative estimate of flux on different surfaces experiencing the same light spectrum, since $\sigma$ will be constant across these surfaces (for a given mineral).

Sohbati and co-workers expanded this model to study the depth dependence of the bleaching, adding an additional exponential component^16^:

2 $$L={L}_{0}{e}^{-\overline{\sigma {\varphi }_{0}}t{e}^{-\mu x}}$$

where $\mu$ (mm^−1^) is the attenuation coefficient for incident light and $x$ (mm) is the depth below the exposed surface. This model assumes that bleaching occurs in a narrow spectral band, hence wavelength dependence can be neglected for simplicity.

In principle, if we could map a high-resolution spatial distribution of luminescence (metastable states) in a cavity using OSL, it would allow us to derive the light flux that resulted in such a distribution, using Eqs. (1 and 2). Unfortunately, OSL is a highly inefficient process where one trapped electron gives rise to one photon, by electron–hole annihilation, making high-resolution imaging of the distribution of metastable states impractical because of low signal-to-noise ratio. Prasad et al. demonstrated a novel way to measure the principal trap in feldspar, avoiding this electron–hole recombination process, by selectively exciting the electron between the ground and the excited state of the principal trap, and detecting the Stokes-shifted radiative relaxation^20^. This infrared photoluminescence (IRPL) emission has two emission peaks at 880 and 955 nm^21^. Unlike OSL, IRPL does not involve electron–hole recombination; instead, the signal originates from repeated transitions of the trapped electrons (in the principal trap in feldspar) between excited and ground states of the defect, i.e. without the destruction of the metastable state^20–23^. Therefore, a single trapped electron can produce millions of photons compared to only one in OSL, resulting in an large increase of the luminescence signal which permits high resolution, non-destructive imaging. Thus, IRPL is a very powerful method in which one electron can give rise to an unlimited number of photons, and thus it makes it possible to obtain a precise (high sensitivity), non-destructive measurement of the spatial distribution of the principal trap. As discussed before, the spatial variations in the relative proportions of destroyed versus pristine metastable states (principle trap) can then help us to derive the light flux using Eqs. 1 or 2.

Using the metastable states in feldspar and non-destructive IRPL measurements, we estimate light flux in artificial cracks of various widths (0.05, 0.3 and 1 mm), which have been exposed to a solar simulator for a period of up to 30 days. We measure the IRPL periodically over this exposure period to estimate spatially-resolved rates of bleaching of the principle trap. Subsequently, we use these measurements to derive light flux distributions along and across these cracks. Our experimental design specifically targeted cracks in rocks in order to highlight both the novelty of our approach and the enormity of the challenge. The proposed method could be adapted to study light propagation in different cavity forms, using monochromatic or broadband light sources, and using natural or artificial (doped insulators) light sensitive sensors.

## Results

**Figure 1:**
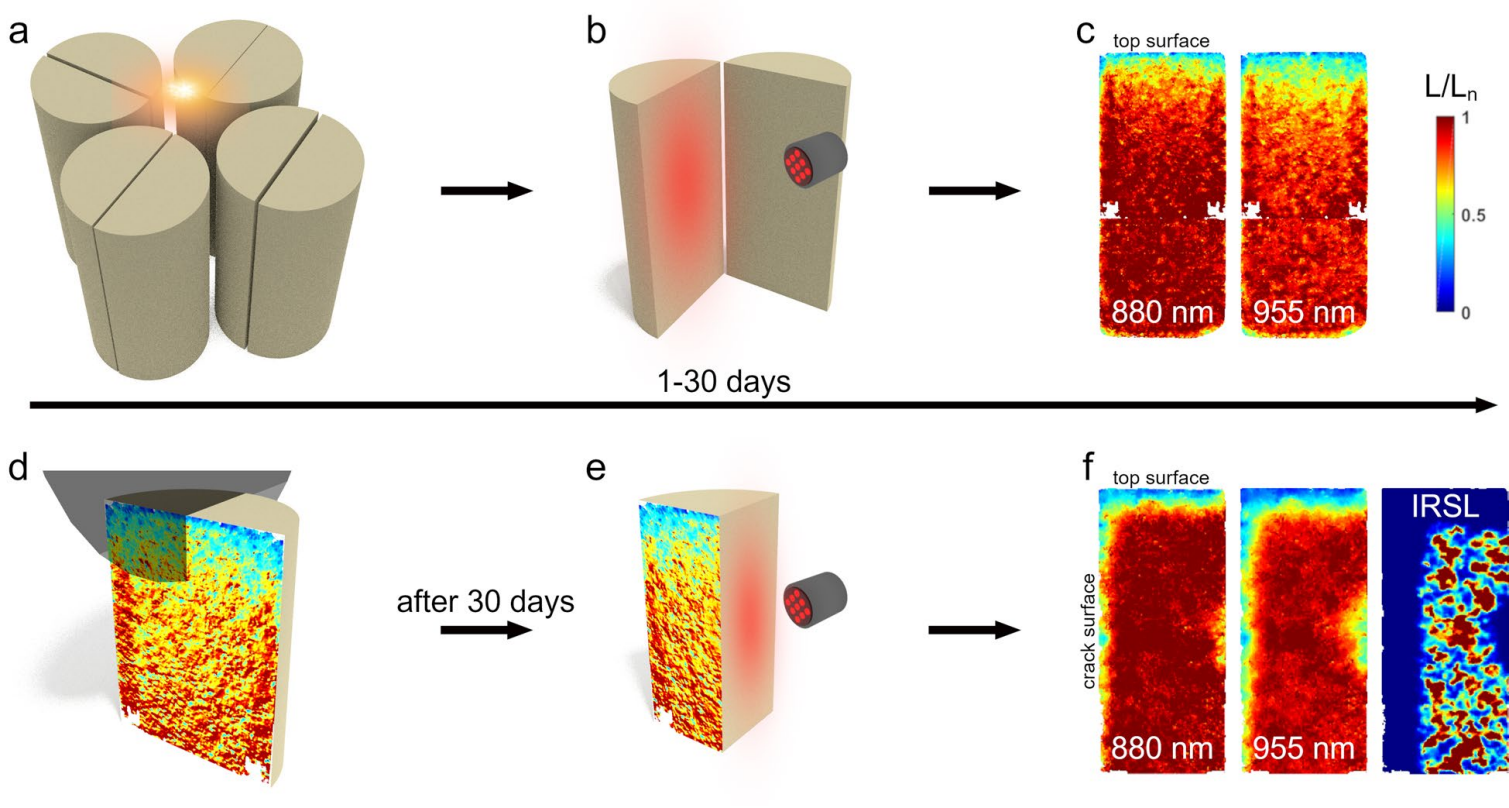
Schematic presentation of the measurement procedure. (**a**) Four artificially cracked cores positioned tightly under a solar simulator. (**b**) IRPL imaging of the inner crack surface during a period of 1–30 days. (**c**) IRPL_880_ and IRPL_955_ ratio maps of the crack surface for a 0.05 mm crack width after 10 days of illumination. (**d**) Schematic representation of the cutting of a core half perpendicular to the crack surface, after 30 days of illumination. (**e**) IRPL imaging of the perpendicular cut surface. (**f**) IRPL_880_, IRPL_955_ and IRSL ratio maps of the perpendicular cut surface for a 1 mm crack after 30 days of illumination. Identical scale bar as in (**c**).

Four rock cores were first irradiated with gamma rays to create trapped electrons (mapped using IRPL), and then cut in half along the axis. Cracks of different widths were simulated by placing spacers between the two halves. All these steps were done under dark conditions. The ‘cracked’ rock cores were then positioned under a solar simulator as shown in Figs. 1a and S1. To investigate the dependence of crack orientation on irradiance^9,24^, three cracks (0.05, 0.3 and 1 mm) were positioned radially outwards of the center of the light illumination allowing all the light to enter directly into the crack, whereas, the fourth core with a crack width of 1 mm was positioned perpendicularly to its 1 mm crack counterpart. In the latter case we expect less direct light, compared to the other three cases, due to shadowing by one half of the core. High-resolution 2D IRPL^25–27^ images were taken of the complete crack inner surface in-between light exposure intervals allowing us to track the progression of light induced destruction of the metastable states (bleaching) over time (Fig. 1b). The core halves were removed every 24 h during the first 10 days (as well as after 20 and 30 days) for measurements of the 2D IRPL signal ($L$) at both 880 and 955 nm, on each half. Luminescence ratio maps (${L/L}_{n}$) displayed in Fig. 1c were attained by dividing the measured values by their corresponding luminescence prior to any light illumination (details can be found in the Materials and Methods section). For all further luminescence profile analysis, the IRPL_955_ signal was chosen, as it bleaches faster compared to the IRPL_880_ signal, and hence offers a larger dynamic range of intensities for our investigations. For this group of measurements the time-dependent model presented by Eq. (1) was used to estimate the light flux at any position on the crack surface.

After the 30 days of solar simulator illumination, a cut was made on one of each of the core halves, along a plane perpendicular to the crack surface as well as the core surface (Fig. 1d), in order to use the depth-dependent model showed in Eq. (2) to estimate light flux at any position along the crack surface. Subsequently, the same 2D IRPL measurements were performed on these newly created perpendicular surfaces to the crack (Fig. 1e). Furthermore, infra-red stimulated luminescence (IRSL), was performed at the end of the experiment, i.e. 30 days exposure. This variant of the OSL technique could not be measured during the experiments to observe the time evolution of trapped electrons, since it is a destructive measurement technique. Therefore, both IRPL 880 and 955 nm as well as IRSL ratio maps are created as shown in Fig. 1f after the 30 day exposure period. These luminescence maps reflect bleaching of luminescence as light travels from air into the rock mass (i) at the top surface of the core, and (ii) sideways from crack into the rock. Therefore, one can extract luminescence-depth profiles from the top exposed surface as well as at various depths down the crack surface to derive light fluxes using Eq. (2).

Hereafter we refer to luminescence results in the two categories as per the measured plane: a) along the **crack surface**, where IRPL is measured as function of time over a 30 days exposure period, and b) along the **perpendicular surface**, where IRPL and IRSL signals are measured as a function of depth from the crack surface, at the end of 30 days exposure.

### Light orientation dependency

**Figure 2:**
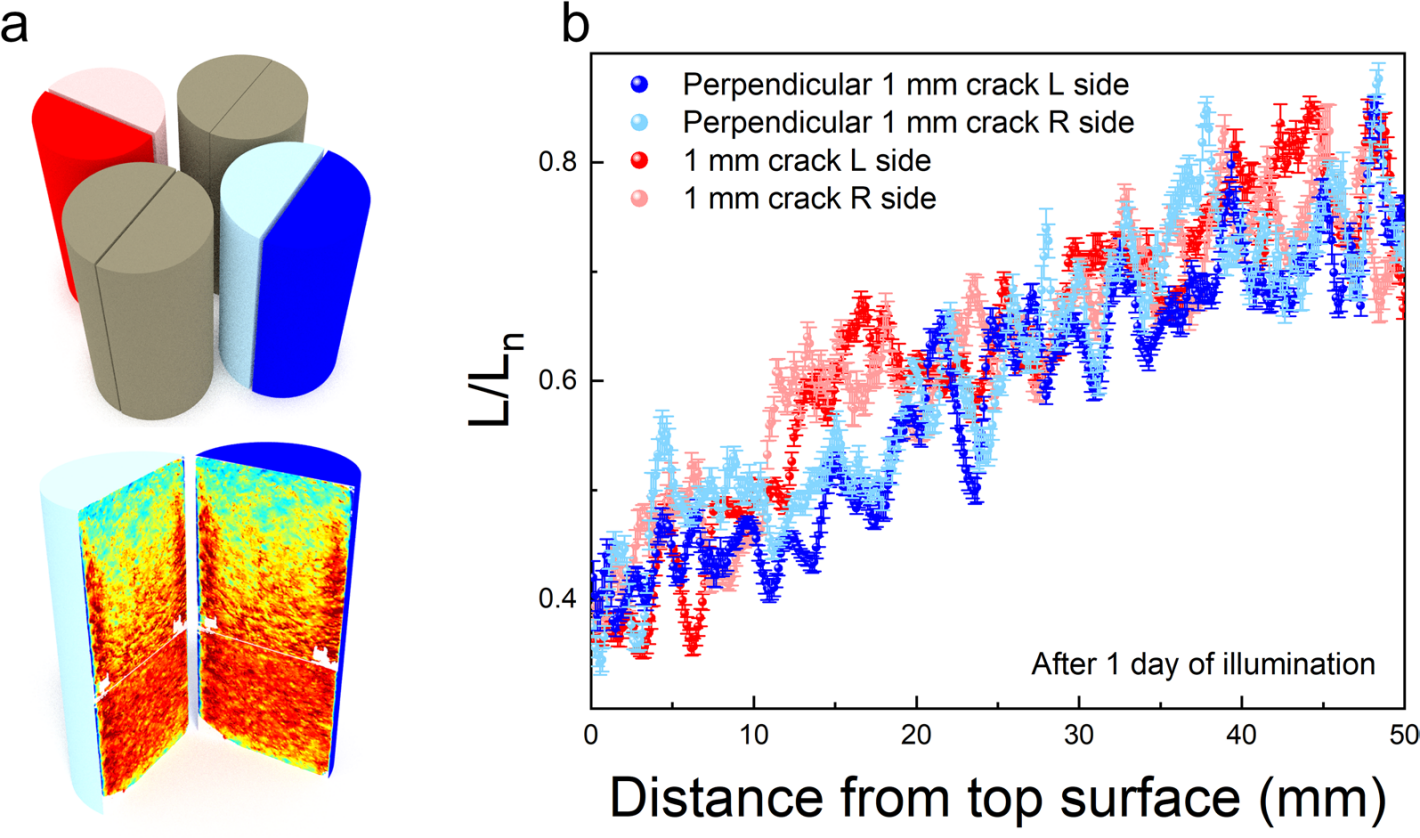
Effect of crack orientation. (**a**) Schematic representation of the two cores with 1 mm crack widths (blue and red) positioned perpendicularly to each other. (**b**) Crack surface bleaching profiles of both halves of the two perpendicular cores.

The very first goal was to investigate the possible effect of shadowing on the crack surfaces depending on the orientation of the cores with respect to the light source in the solar simulator. Here, we compared data from the two cores with identical cracks (width = 1 mm), placed perpendicular to each other (Fig. 2a). Bleaching profiles along the crack plane were constructed (see Materials and Methods section and Figure S2) for the two halves of each core and plotted in Fig. 2b. As may be expected, the crack plane profiles from the two halves of the same core are indistinguishable (Fig. 2b). We see only minor local divergences e.g., around 15 mm and 40 mm depth, between the luminescence profiles of the pairs of crack planes oriented perpendicular to each other. Since these differences are not systematic as a function of distance, we infer that they are likely caused by random local variations in mineral composition in the two cores. Based on these results, only one side of each core is reported for further analysis. We further conclude that there is negligible effect of possible shadowing or orientation of the cores (radial vs. perpendicular) on luminescence bleaching, which in turn suggests that luminescence bleaching must mainly be caused by diffused/scattered light which will be discussed in detail later.

### Time-dependent luminescence profiles along the crack plane

**Figure 3:**
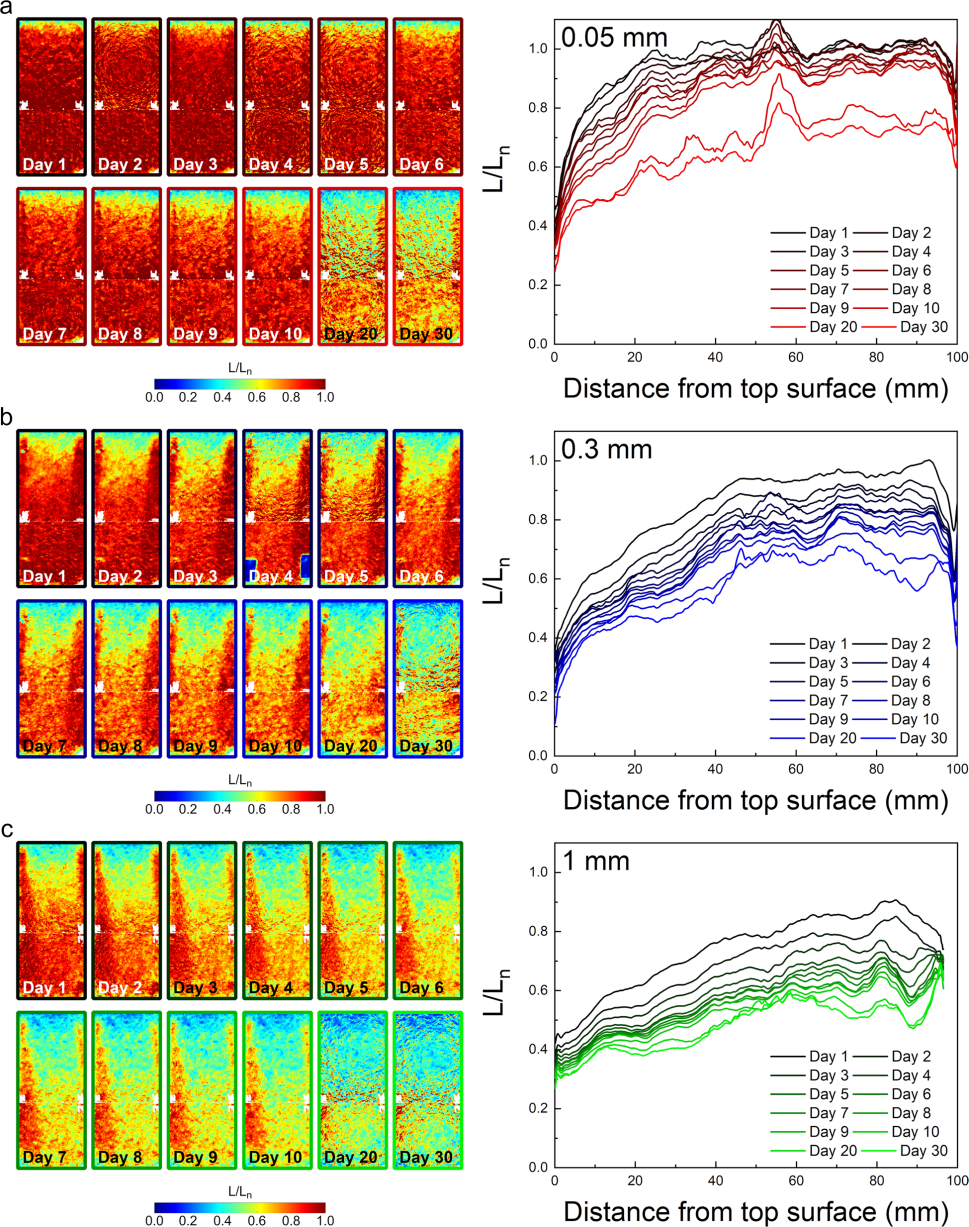
(Left column) Normalized IRPL_955_ maps along the crack surface and the corresponding luminescence profiles as a function of distance down the crack. The data are shown for the three different crack widths (**a** red) 0.05, (**b** blue) 0.3 and (**c** green) 1 mm. (Right column) The right side of each figure shows the normalized IRPL_955_ signal as a function of the distance from the top surface.

All IRPL_955_ ratio maps for the 0.05, 0.3 and 1 mm crack widths are shown in Fig. 3a–c, respectively. The red regions show the filled traps, whereas blue regions show the empty (bleached traps). As expected, the bleaching front progresses systematically over the 30 day bleaching period. A negligible drop in the luminescence ratio appearing in a thin layer at the bottom (Fig. 3a) is due to light leaking into the cracks from the bottom surface, since we did not actively isolate possible light leakage from the bottom. Similarly, we observe that the bleaching progresses in a U shaped zone in the middle of the crack; this effect occurs because we had tightly isolated the sides of the cores. Both these unexpected effects attests to the sensitivity of our method to minor variations in light flux—as we also find in our comparisons of flux for the three crack widths.

The right hand panels in Fig. 3 show corresponding distance-dependent luminescence bleaching profiles derived from the corresponding luminescence images (left panel) for each crack. The data was integrated only in the middle of each image to avoid the effect of light blocking from the sides. Each curve corresponds to one image and each figure shows bleaching profiles for all days ranging from 1 to 30. After the first day of light exposure (black curve), a clear difference in the bleaching extent can be observed across the crack widths. For the thinnest crack width, an almost sigmoidal shape luminescence profile is obtained with significant bleaching along the crack towards the top of the core, and undetectable bleaching below ~ 40 mm depth in the core. This bleaching trend is comparable to a previously reported typical luminescence-depth profiles below rock surfaces^16^, suggesting that light is attenuating exponentially down the crack. However, in the case of the larger crack widths, significant luminescence bleaching occurs along the crack plane at all depths down the surface of the core. The data shows a systematic gradual increase in luminescence down the core surface. After 30 days of light exposure, (full color line) luminescence is substantially bleached throughout the crack plane for each crack width. Nevertheless, the bleaching intensity ranges from, for example, 40% to 65% (at 25 mm distance from the top surface) for the 0.05 mm to the 1 mm crack width.

**Figure 4:**
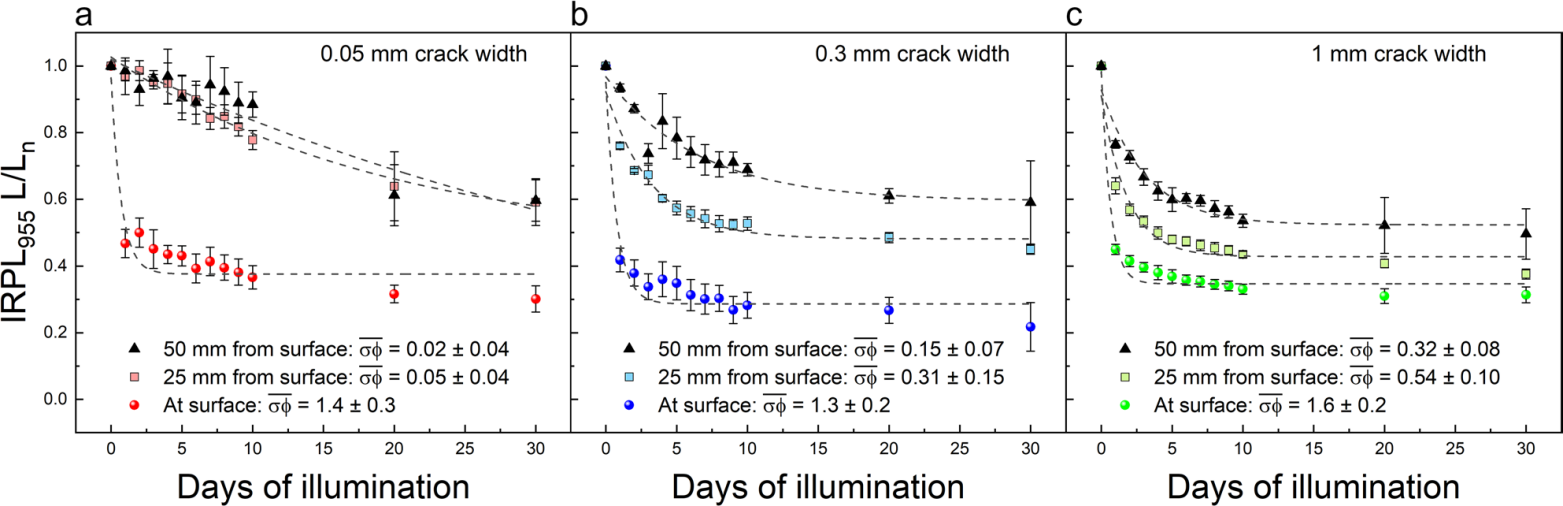
Dependence of normalized IRPL_955_ values at the crack surface on the exposure duration. Values were extracted at 0, 25 and 50 mm distances from the top surface for the (**a**) 0.05, (**b**) 0.3 and (**c**) 1 mm crack widths. Dashed curves show the best fit of Eq. (1) to the data.

To derive the flux distribution along the crack surface, time dependence of luminescence was analyzed at selected depths from the top of the core. Three areas, each with 2 mm width, were chosen (see Fig. S3): at about ~ 0, 25 and 50 mm depths down the crack. The average luminescence ratio values in these regions were extracted and plotted as a function of time for each crack width (Fig. 4). The surface (0 mm depth), of all three cracks have indistinguishable luminescence decay rates. This is expected since bleaching is dominated by light arriving at the top surface of the core and the flux should be identical for all three samples irrespective of crack width. In addition, this confirms that the photoionisation cross section $\sigma$ is alike for all three cores, as one would expect. A comparison of bleaching rates at different depths suggests the decay of luminescence is most rapid near the surface and decreases systematically down the crack both as a function of depth and crack width. These data confirm that light attenuation down the crack depends on the width of the crack; the larger the crack width the more rapid the luminescence decay.

Using the time-dependent model function (Eq. 1), values for the effective decay rate of the luminescence were calculated for each curve (these are displayed in the legends of Fig. 4). The $\overline{\sigma {\varphi }_{0}}$ is consistent with zero already at 25 mm from the top surface in the 0.05 mm crack (0.05 ± 0.04), whereas, for the larger cracks (0.3 and 1 mm) visible in Fig. 4b, c, $\overline{\sigma {\varphi }_{0}}$ values are 0.31 ± 0.15 and 0.54 ± 0.10, respectively. Similar trend is observed in data at 50 mm depth; here the 0.05 mm crack has a decay rate of only 0.02 ± 0.04, compared to those of 0.15 ± 0.07 and 0.32 ± 0.06 for the 0.3 and 1 mm cracks, respectively. While the increase in bleaching rate (and hence flux, if $\sigma$ is constant) with crack width is expected, interestingly, these quantities do not seem to vary linearly with crack width, an aspect discussed later in detail.

### Depth-dependent luminescence profiles along a surface perpendicular to the crack

**Figure 5:**
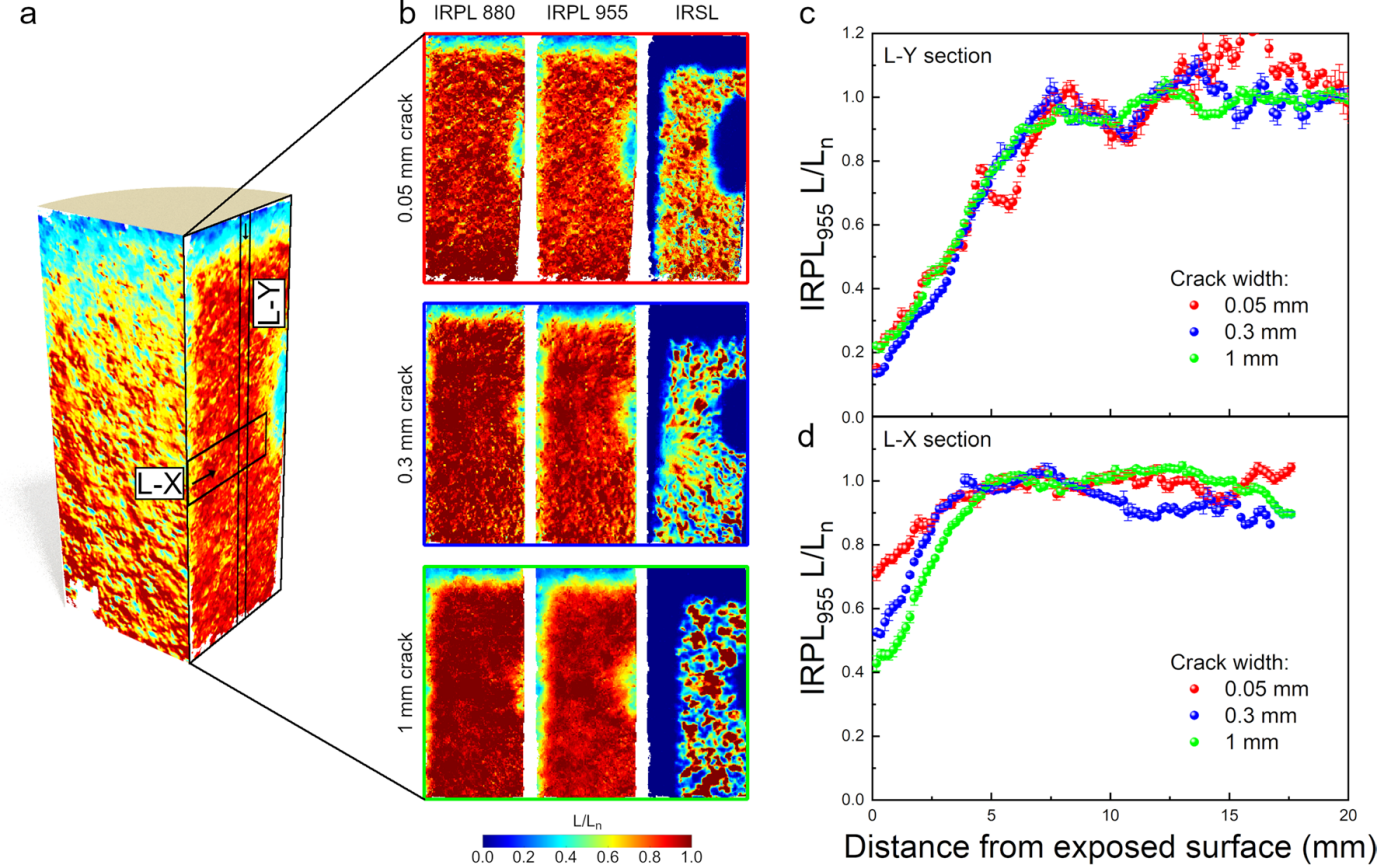
Luminescence measurements on the perpendicular cut surfaces. (**a**) Schematic representation of the perpendicular cut. (**b**) IRPL_880/955_ and IRSL maps of the perpendicular cuts of different crack widths. (**c**) Depth profiles taken from the IRPL_955_ luminescence values from the top surface (L-Y section). (**d**) Depth profiles taken from the crack surface (L-X section) at a distance of 25 mm from the top. The data is shown for the different crack widths.

As discussed previously, an alternative method to estimate the dependency of flux values at different positions in the crack is to analyze luminescence-depth profiles (L-X) using Eq. 2. For this analysis we used profiles from the planes cut perpendicular to the crack surface (Fig. 5a) after 30 days of exposure. IRPL_880_ and IRPL_955_ and IRSL ratio maps obtained from these sections are shown in Fig. 5b for each crack width. Unfortunately, we observed a bleached half circle on the IRPL and IRSL maps, which is in fact due to imperfect isolation of the sides of the core leading to unexpected light leakage. This unexpected behavior again attests to the sensitivity of our method. Nevertheless, this anomaly did not have any impact on our calculations and conclusions as we could easily discard this region for further analysis.

As mentioned beforehand, these luminescence maps reflect bleaching of luminescence from both top surface and light entering sideways from crack into the rock. This is manifested as a continuous bleached zone extending from top to the side of the crack. However, it is important to distinguish between these two processes, since bleaching from the top is not a function of the crack width, while bleaching from the side should depend upon the crack width based on the data reported in the previous section.

For detailed analysis, we first derived luminescence-depth profiles for bleaching occurring from the top surface (L-Y section in Fig. 5a). Thin 5 mm wide segments were defined at the center of the luminescence ratio maps where we are certain that there is no bleaching occurring from the crack surface or sides of the core. These luminescence ratio values are plotted as function of distance from the top surface for all three crack widths in Fig. 5c. As expected, all these L-Y profiles align with each other irrespective of crack width, since bleaching is due to light exposure of the top surface of the core and not through the cracks themselves.

Having confirmed the reproducibility of our approach through the measurements above, we then looked at luminescence depth profiles perpendicular to the cracks; here any differences in the profiles should be due to different crack widths. For illustration, profiles at 25 mm depth from the core surface (L-X section in Fig. 5a) were chosen. Figure 5d shows these luminescence-depth profiles for the three different crack widths. The inflection point of the profile (also known as the bleaching front) becomes deeper with increasing crack width because of the increasing light flux in the crack. This is consistent with the predictions of Eq. 2, i.e. profiles get deeper with an increase in the ${\varphi }_{0}$ if $t$ and $\sigma$ are constant. As discussed next, fitting Eq. 2 to such profiles allows alternative evaluation of $\overline{\sigma {\varphi }_{0}}t$, which in turn can be used to estimate the light flux.

To understand the variation in light flux down the crack, L-X profiles were taken from multiple segments of approximately 5 mm in width, at increasing depth from the top surface, as seen in Figure S4. For the IRPL luminescence data, the first 8 mm were disregarded, to avoid any bleaching effects due to light penetration from the top surface (see the top blue bleached zone in Fig. 5a). The values were further normalized by the average luminescence values at 15 mm from the crack surface. In case of the IRSL values down to 13 mm were ignored for plotting L-X profiles, since electron traps giving rise to IRSL bleach must faster than those giving rise to IRPL, and therefore IRSL bleaching effects greater depths compared to IRPL^28,29^.

**Figure 6:**
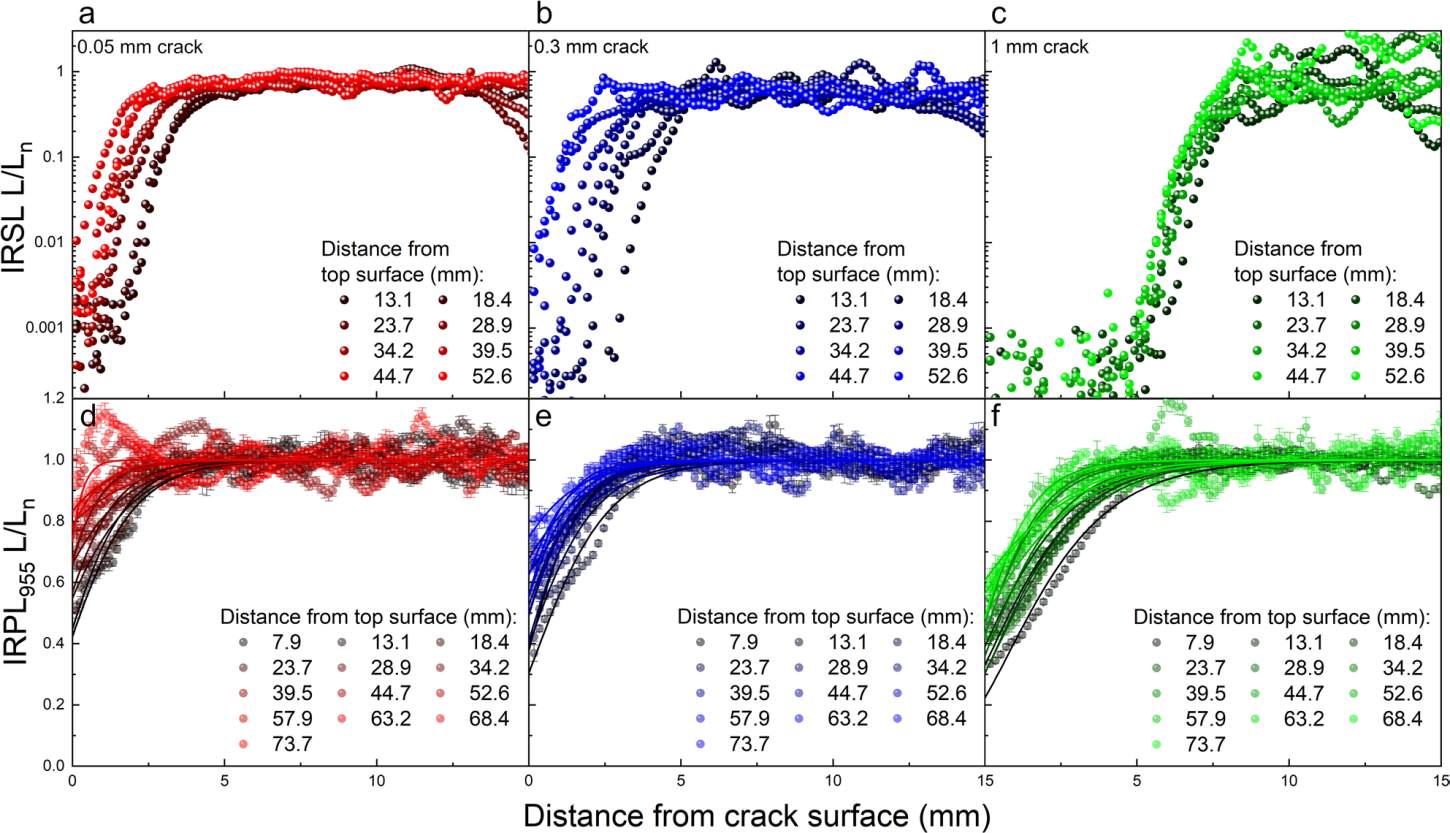
Normalized IRSL- and IRPL_955_-depth profiles acquired from 5 mm L-X sections at various distances from 8 to 80 mm from the top surface for the (**a**) 0.05, (**b**) 0.3 and (**c**) 1 mm crack widths. Solid curves represent the best fits of the data to Eq. (2).

In Fig. 6a–c, the IRSL depth profiles are plotted for the 0.05, 0.3 and 1 mm crack widths, respectively. Because of greater bleaching in the IRSL signal, the profiles are shown on Ln scale^17^ in order to make luminescence levels in the bleaching zone clearly visible. For the 0.05 mm crack (Fig. 6a) a systematic decrease in the depth of IRSL bleaching front (inflection point of the profile), ranging from ~ 2 to 0 mm, can be observed as a function of distance from the top surface.; this suggests a “V” shape bleached region which can be seen in the ratio maps in Fig. 5b. The same is also evident for the 0.3 mm crack, where inflection point ranges between 3 and 0 mm. Thus, we expect significant attenuation of light flux down the crack in both these cases. On the other hand, for the largest 1 mm crack (Fig. 6c), throughout the whole crack an identical depth of bleaching is observed at 5–6 mm suggested a more uniform flux down the crack.

It is important to point out that in all IRSL depth profiles, no matter the crack width or crack depth, the slope at the bleaching front, which is governed by the attenuation coefficient ($\mu$) is very similar, i.e. they all appear parallel. Since $\mu$ is a function of wavelength, this observation strongly suggests that light spectra are the same at all surfaces within the crack, and this similarity is irrespective of the crack width. The reduction in light intensity down the crack must therefore be uniform for all wavelengths.

In Fig. 6d–f, the IRPL_955_—depth profiles are plotted for the 0.05, 0.3 and 1 mm crack widths, respectively. We used these IRPL data to derive flux values by fitting the first order kinetic model (Eq. 2), since the signal-to-noise ratio is much higher for IRPL compared to the IRSL data; this is, as discussed earlier, due to the steady-state (non-destructive) nature of IRPL as opposed to the destructive IRSL measurements. Moreover, using the IRPL_955_ signal for quantitative analysis allows us to compare flux estimates with those obtained from luminescence-time data along the crack surface in the previous section. The plots show L-X profiles for continuous segments ranging from 8 to 74 mm from the top surface. Most of these depth profiles can be well described by Eq. 2, with a few exceptions for the 0.05 mm crack width where almost no bleaching was evident at relatively deeper position down this narrow crack. The decay rates ($\overline{\sigma {\varphi }_{0}}$) and attenuation coefficients ($\mu$) obtained from fitting these data with Eq. 2 are summarised in Figure S5. While the decay rate shows a systematic decrease down the crack surface for each crack width (Figure S5a), $\mu$ shows random fluctuations with depth, largely governed by the goodness of the fit (Figure S5b). Such random fluctuations in $\mu$ arise possibly from slight mineralogical variations, which is often seen in rocks^30^. Nonetheless, a lack of any systematic trend in $\mu$ again confirms our inference based on the IRSL data that there is no significant spectral change in the light when going down the crack. Therefore, to derive absolute flux values we will consider the $\sigma$ value constant at all positions of the crack surface, allowing us to attribute decay rate ($\overline{\sigma {\varphi }_{0}}$) variations down the crack surfaces to light flux variations ($\overline{{\varphi }_{0}}$).

In the previous section, luminescence time-dependant profiles were described at only three positions down the crack (0, 25 and 50 mm, see Fig. 4). Now, this process was repeated for the segments, which transpose the segments used in the L-X profiles (Figure S4) on to the crack plane (Figure S6). Thus, we could directly compare $\overline{\sigma {\varphi }_{0}}$ derived from luminescence-time data with the $\overline{\sigma {\varphi }_{0}}$ values derived from the L-X data on the plane of the crack. The fitting analysis of luminescence-time data in these segments is shown in Figure S7 for each crack width and the $\overline{\sigma {\varphi }_{0}}$ results from this analysis are summarised in Figure S8 as a function of distance down the crack surface. Therefore, Figures S5 and S8 allows us to directly inter-compare flux distribution on the crack surface derived using the two models (Eqs. 1 and 2). To our surprise, different values of $\overline{\sigma {\varphi }_{0}}$ were obtained depending on the model used and these differences are accentuated as we progress towards the top of the crack surface. For instance, at the very top section, $\overline{\sigma {\varphi }_{0}}$ values estimated by fitting Eq. 1 to luminescence-time data (Figure S8), range between 0.2 to 1.5 day^−1^, whereas the values estimated by fitting Eq. 2 to luminescence-depth (L-X) data in the same section range between 0.03 to 0.05 day^−1^, both depending on the crack width. The wider cracks (0.3 and 1 mm) crack show significantly larger divergence in the $\overline{\sigma {\varphi }_{0}}$ values derived from the two models, as compared to the thin crack (0.05 mm).

To understand these differences we need to appreciate the subtle difference between the two models. The time dependent model (Eq. 1) is sensitive to all wavelengths impacting the crack surface, whereas the depth dependent model is sensitive to the wavelengths that effectively penetrate the rock mass. Thus, the differences we see between the two models can be reconciled with strong absorption of the light UV component at the crack surface, which has high interaction cross section for trapped electrons^31,32^. And although the light spectrum on the crack surface remains unchanged, based on lack of any systematic difference in the attenuation coefficients down the crack surface in either IRSL or IRPL data (discussed earlier), there occurs a change in the light spectrum as it traverses from the surface of the crack into the rock mass. Based on this comparison, we infer that UV light dominates bleaching at the crack surface (luminescence-time data), whereas bleaching into the rock (L-X profiles) is dominated by visible-NIR light.

Since our data does not show any change in the light spectrum on the crack plane it can be easily shown that the variation in the decay constant is equivalent to the variation in flux. Following Sohbati et al.^16^, the decay constant can be defined as:

3 $${(\overline{\sigma {\varphi }_{0}})}_{y}=\int \sigma \left(\lambda \right){{\varphi }_{0}\left(\lambda \right)}_{y}d\lambda$$

where $\lambda$ is the wavelength and $y$ is the position down the crack. The ratio of decay constants at two given depths $y0$ and $y$ ($y$ > 0) is:

4 $$\frac{{\left( {\overline{{\sigma \varphi_{0} }} } \right)_{y0} }}{{\left( {\overline{{\sigma \varphi_{0} }} } \right)_{y} }} = \frac{{\smallint \sigma \left( \lambda \right)\varphi_{0} \left( \lambda \right)_{y0} d\lambda }}{{\smallint \sigma \left( \lambda \right)\varphi_{0} \left( \lambda \right)_{y} d\lambda }}$$

If there is no change in the light spectrum then,

5 $$\varphi_{0} \left( \lambda \right)_{y} = k_{y} \varphi_{0} \left( \lambda \right)_{y0}$$

where $k_{y}$ is a constant less than unity. Substituting this relation in the Eq. (4) gives allows us to estimate $k_{y}$ from our measurements:

6 $$k_{y} = \frac{{\left( {\overline{{\sigma \varphi_{0} }} } \right)_{y} }}{{\left( {\overline{{\sigma \varphi_{0} }} } \right)_{y0} }}$$

Thus, the value of flux at $y$ with respect to $y0$ is:

7 $$\varphi_{0} \left( \lambda \right)_{y} = \frac{{\left( {\overline{{\sigma \varphi_{0} }} } \right)_{y} }}{{\left( {\overline{{\sigma \varphi_{0} }} } \right)_{y0} }}\varphi_{0} \left( \lambda \right)_{y0}$$

**Figure 7:**
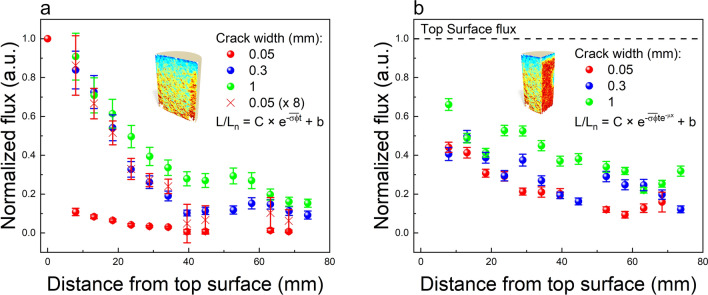
Dependency of light flux down cracks of various widths. Flux normalized by the decay rates at the surface for the (**a**) time-dependent and (**b**) depth-dependent model.

Equation 7 provides the relative distribution of light flux down the crack. The absolute light flux distribution can be derived if we know the absolute value of flux on at least one of the depths, for example by direct measurement on the top surface or by calibration using a measured response function of luminescence decay constant ($\sigma \varphi$) to flux ($\varphi$). In Fig. 7 we utilize Eq. (7) to compare our two models, by normalizing the flux values by the flux estimated near or at the top surface. In Fig. 7a top surface flux was calculated from Eq. (1) using the top 3 pixels of the crack surface; while not exact, we consider that this measurement closely represents the flux at the top surface of the rock. On the other hand, in Fig. 7b, the top surface flux was calculated by fitting the L-Y profiles (Figure S9).

While the normalized flux values, in Fig. 7a, of the larger crack widths (0.3 and 1 mm) align on top of each other, the 0.05 mm crack (red dots) shows a rapid, likely exponential, drop by an order of magnitude within the first 5 mm. However, if we ignore the very first point, the distribution of flux is the same for all three crack width, which is clearly evident when multiplying the values for the 0.05 mm crack with a factor of 8 (red cross). In Fig. 7b, all 3 crack widths have the same flux distribution and a much less disparity in normalized flux values (only a factor of 2 between the 0.05 and 1 mm crack). The inter-comparison of Fig. 7a, b, suggests that the sharp flux drop observed for 0.05 mm crack in Fig. 7a, must be related to the presence of the UV component near the rock surface which is quickly absorbed as light moves down the crack or into the body of the rock. In addition, the trends in Fig. 7a, b seem broadly similar except in the first ~ 10–20 mm from the top surface; this again suggests that the UV component near the rock surface impacts model 1 much more than model 2 because of its surficial bleaching effect (i.e., UV will induce bleaching at the surface but not at depth due to strong absorption).

## Discussion and outlook

We find it remarkable, especially for the light flux derived from the L-X profiles, that a 20 times smaller crack width has about half of the light flux going down the crack. It seems that most of the bleaching, especially into the core, is due to diffused or scattered light, which in fact is not drastically different between cracks of different width. In addition, confirming the previous conclusion that crack orientation does not have a significant influence on bleaching. This observation has important implications for rock surface exposure dating, suggesting that geometrical effects are far less important than believed so far^33^. Furthermore, our method can help resolve the role of direct solar insolation in governing moisture-induced crack propagation^10^ by enabling quantification of light flux distribution in cracks as a function of their orientation. Our preliminary data suggest that orientation does not significantly change the light flux, likely because of the dominance of diffused light in the crack.

It is important to examine our assumption of wavelength independency of the light flux, since this is crucial for estimating the light flux values. OSL excitation spectra in these feldspar show an exponential dependence of trap eviction on excitation energy with an additional NIR resonance at about 850 nm^23^; hence, bleaching effectively occurs in relatively narrow spectral window(s), e.g. UV-blue-green in quartz and to some extent an additional NIR window (around 850 nm) in feldspar. Measured luminescence-depth profiles in ubiquitous natural minerals such as quartz and feldspar show a good fit to the model which assumes that light spectrum does not vary with depth^17^. Finally, the invariance of slope in the IRSL-depth profiles (Fig. 6), and a non-systematic variation of estimated $\mu$ values with crack depth obtained from IRPL_955_ (Figure S5b) again support the assumption that there are no significant systematic removal of short wavelengths for the size of cavities examined here. Therefore, we believe that to the first order our assumption of invariant light spectrum is valid. Nevertheless, the possibility of wavelength dependence of the daylight flux or attenuation constant should be kept in mind when dealing with natural systems, and further should quantify larger order effects and estimate any uncertainties arising from these.

The technique developed here can be applied to a wide range of applications that involve light flux measurements in challenging scenarios especially in natural environments. For example, it could be interesting to examine how light flux distribution in cavities or even the surfaces of rocks relate to algal/microbial growth. Similarly, one could map light fluxes using naturally exposed rocks in forest covers and relate these to growth of vegetation. The ability to measure light flux from mapping luminescence within and across the cracks combined with the relative insensitivity of light flux to crack width, raises for the first time the possibility of obtaining chronologies of when fractures formed in the past (dating cracks). The results obtained in our study (Fig. 7) suggest that such fracture dating can be accomplished by applying the existing principles of luminescence exposure dating to fractures.

Our novel method could also be applied to historical building stones or concrete for detection of micro fractures. Such materials indeed contain feldspar and a relative change in luminescence emission from fractured vs un-fractured concrete would yield information of not only the existence of the fractures but also how old such fractures are, thus providing critical information on the durability of these materials. Since metastable states are easily bleached by light within a matter of seconds to hours, our method can also be applied for detection of fracture zones by artificial illumination.

We focussed on feldspar since it is the most common mineral in Earth’s crust and therefore widely applicable. However, many artificial materials, much more sensitive than feldspar or with different energy structure for the electron trap, could be used for laboratory based applications to obtain a fundamental understanding of light transmittance in cavities. For further method developments, our study can be repeated with monochromatic lights to study the exact dependence of light transmittance in the cavity on wavelength.

## Conclusions

We have developed a novel, passive light sensing technique for high-resolution mapping of light flux in ultra-thin cavities. Since this technique is based on mapping of the atomic scale, light-sensitive metastable states (trapped electrons), there is, for all practical purposes, no minimum constraint to the size of the cavity. The method can also be applied to estimate flux distributions on exposed surfaces at a high spatial resolution.

We demonstrate the application of our technique to the measurement of light flux in artificial cracks of 0.05, 0.3 and 1 mm widths, exposed to solar simulator for a period of 30 days. Contrary to our expectation, the modelling of the spatio-temporal evolution of the metastable states suggests that light flux at the crack surfaces does not scale with the crack width, and is in fact almost independent of crack widths > 0.3 mm. Furthermore, the orientation of the crack does not have a significant influence on the light induced decay of the metastable states. These observations suggest that light propagation within cracks in nature must be dominated by diffused light, and therefore direct solar insolation is not an important factor in controlling the moisture content and hence subcritical crack growth in rocks. These novel findings raise a unique possibility of dating crack formation in rocks to obtain insights on how fractures form in response to climate change.

Our technique has the potential to bridge the knowledge gap in a wide range of areas ranging from the role of crack propagation in landscape evolution (geomorphology) to identification of cracks in concrete, ceramics and materials for radioactive waste disposal, to understanding linkages between light flux and biota in rocks and cavities.

## Materials and methods

### Rock cores preparation

The rock samples chosen for this experiment were pale white granites, collected from an unknown location in China. The feldspar composition was predominantly K-rich, and gave bright IRPL and IRSL, which was also easily reset via optical stimulation.

Four cores of about 5 cm in diameter and 10 cm in depth were drilled perpendicular to the rock surface. Subsequently they were cut in half, perpendicular to the surface, to create the artificial cracks. The cores were first heated to remove all naturally trapped charges and later given 5 kGy gamma irradiation to create a homogeneous distribution of trapped charges in the entire core for normalization purpose. IRPL of the initial dose was measured and used for normalization of the subsequent data. The pairs of core halves were then positioned next to each other, separated by spacers of either 0.05, 0.3 or 1 mm thickness, placed at the four corners of the crack plane. Lastly, the samples were encapsulated by light-tight foil tape to eliminate any unwanted bleaching, allowing only the top surface to be exposed to light.

### Solar simulator bleaching

A Honle SOL 2 sunlight test chamber was used for illuminating the rock cores. A light spectra, similar to the natural sunlight is produced by a metal halide bulb. The solar simulator generates an intensity of 910 W/m^2^, which is 6.5 times higher than that of outdoors sunlight and can evenly irradiate a total test area of 1400 cm^2^. All four cores were positioned in this area to obtain the same amount of light illumination throughout the experiment.

### IRPL imaging and processing

The core halves were measured in the dark using an EMCCD (electron multiplying charged coupled device)-based system, seen in Figure S10 and described in detail in Sellwood et al.^27^ 2D IRPL signals ($L$) for both 880 and 955 nm emissions were measured using an 830 nm collimated beam, reshaped to attain a near circular power distribution of 0.8 mW/cm^2^ over a sample area of 8 × 8 cm^2^. The images were analyzed using a imaging process toolbox in MATLAB, where they were aligned, cropped and divided by their corresponding initial luminescence values ($L_{n}$) measured after the given gamma dose of 5 kGy from the cobalt source to normalize for spatial variations in sensitivity. This resulted in ratio maps ($L/L_{n}$) displayed in the main text figures. Each crack surface was imaged in 2 parts due to the areal coverage restriction of our imager. The respective images were correlated using the black marker on the crack surface. The black marks emit no luminescence, and therefore appear as white spots after our image analysis.

### Luminescence data analysis

For data analysis, luminescence values are taken from a rectangle segment of the ratio maps, averaging pixel values in one direction in dependence of distance in the perpendicular direction. For instance, in the case of the time-dependent measurements, we took an approximately 5 mm wide rectangle positioned in the middle of the crack surface extending from the top surface (directly exposed to light) to the bottom of the core (Figure S2). A narrow segment was chosen, not to be affected by the shading of the isolating tape around the cores as well as the spacers at the edges.

## Supplementary Information

Supplementary Figures.

## Data Availability

All data generated or analyzed during this study are included in this published article (and its Supplementary Information files).

## References

[1] Roxby DN (2020). Enhanced biophotocurrent generation in living photosynthetic optical resonator. Adv. Sci..

[2] Ren L (2020). Fabrication and cavity-size-dependent photocatalytic property of TiO_2_ hollow nanoparticles with tunable cavity size. Mater. Res. Bull..

[3] Coles DM (2014). Strong coupling between chlorosomes of photosynthetic bacteria and a confined optical cavity mode. Nat. Commun..

[4] Sáez-Blázquez R, Feist J, Romero E, Fernández-Domínguez AI, García-Vidal FJ (2019). Cavity-modified exciton dynamics in photosynthetic units. J. Phys. Chem. Lett..

[5] Soga, N., Spetzler, H. & Mizutani, H. Comparison of single crack propagation in lunar analogue glass and the failure strength of rocks. *Proc. 10th Lunar Planet. Sci. Conf.* **3**, 2165–2173 (1979).

[6] Mizutani, H., Spetzler, H., Getting, I., Martin, R. J. III & Soga, N. The effect of outgassing upon the closure of cracks and the strength of lunar analogues. *Proc. 8th Lunar Sci. Conf.* **59**, 1235–1248 (1977).

[7] Eppes MC (2018). Rates of subcritical cracking and long-term rock erosion. Geology.

[8] Eppes MC, Keanini R (2017). Mechanical weathering and rock erosion by climate-dependent subcritical cracking. Rev. Geophys..

[9] Moores JE, Pelletier JD, Smith PH (2008). Crack propagation by differential insolation on desert surface clasts. Geomorphology.

[10] McFadden LD, Eppes MC, Gillespie AR, Hallet B (2005). Physical weathering in arid landscapes due to diurnal variation in the direction of solar heating. Bull. Geol. Soc. Am..

[11] Nat Bowers. World’s smallest ambient light sensor for wearables. https://www.electronicspecifier.com/products/sensors/world-s-smallest-ambient-light-sensor-for-wearables (2015).

[12] Odom IE, Doe TW (1976). Nature of Feldspar-Grain Size Relations in Some Quartz-Rich Sandstones. SEPM J. Sediment. Res..

[13] Huntley DJ, Godfrey-Smitht DI, Thewalf ML (1985). Optical dating of sediments. Nature.

[14] Erfurt G, Krbetschek MR, Bortolot VJ, Preusser F (2003). A fully automated multi-spectral radioluminescence reading system for geochronometry and dosimetry. Nucl. Instrum. Methods Phys. Res. Sect. B Beam Interact. Mater. Atoms.

[15] Guralnik B (2015). OSL-thermochronometry of feldspar from the KTB borehole, Germany. Earth Planet. Sci. Lett..

[16] Sohbati R, Jain M, Murray A (2012). Surface exposure dating of non-terrestrial bodies using optically stimulated luminescence: A new method. Icarus.

[17] Sohbati R, Murray AS, Chapot MS, Jain M, Pederson J (2012). Optically stimulated luminescence (OSL) as a chronometer for surface exposure dating. J. Geophys. Res. Solid Earth.

[18] Sohbati R, Murray AS, Jain M, Buylaert JP, Thomsen KJ (2011). Investigating the resetting of osl signals in rock surfaces. Geochronometria.

[19] Riedesel S (2019). Optical determination of the width of the band-tail states, and the excited and ground state energies of the principal dosimetric trap in feldspar. Radiat. Meas..

[20] Prasad AK, Poolton NRJ, Kook M, Jain M (2017). Optical dating in a new light: A direct, non-destructive probe of trapped electrons. Sci. Rep..

[21] Kumar R, Kook M, Murray AS, Jain M (2018). Towards direct measurement of electrons in metastable states in K-feldspar: Do infrared-photoluminescence and radioluminescence probe the same trap?. Radiat. Meas..

[22] Kook M, Kumar R, Murray AS, Thomsen KJ, Jain M (2018). Instrumentation for the non-destructive optical measurement of trapped electrons in feldspar. Radiat. Meas..

[23] Sellwood EL (2019). Optical bleaching front in bedrock revealed by spatially-resolved infrared photoluminescence. Sci. Rep..

[24] Génin FY, Salleo A, Pistor TV, Chase LL (2001). Role of light intensification by cracks in optical breakdown on surfaces. J. Opt. Soc. Am. A.

[25] Sellwood EL, Kook M, Jain M (2022). Rapid in situ assessment of luminescence-bleaching depths for deriving burial and exposure chronologies of rock surfaces. Quat. Geochronol..

[26] Sellwood EL, Kook M, Jain M (2022). A 2D imaging system for mapping luminescence-depth profiles for rock surface dating. Radiat. Meas..

[27] Sellwood EL, Kook M, Jain M (2022). Investigating the potential of rock surface burial dating using IRPL and IRSL imaging. Radiat. Meas..

[28] Jain M, Kumar R, Kook M (2020). A novel coupled RPL/OSL system to understand the dynamics of the metastable states. Sci. Rep..

[29] Riedesel S (2021). Site-selective characterisation of electron trapping centres in relation to chemistry, structural state and mineral phases present in single crystal alkali feldspars. J. Phys. D. Appl. Phys..

[30] Ou XJ, Roberts HM, Duller GAT (2022). Rapid assessment of beta dose variation inside cobbles, and implications for rock luminescence dating. Quat. Geochronol..

[31] Wintle AG (1994). Infrared-stimulated luminescence dating of sediments. Radiat. Meas..

[32] Kumar R, Kook M, Jain M (2020). Understanding the metastable states in K-Na aluminosilicates using novel site-selective excitation-emission spectroscopy. J. Phys. D. Appl. Phys..

[33] Gliganic LA, Meyer MC, Sohbati R, Jain M, Barrett S (2019). OSL surface exposure dating of a lithic quarry in Tibet: Laboratory validation and application. Quat. Geochronol..

