# Supplementary material for: Passive atomic-scale optical sensors for mapping light flux in ultra-small cavities
Source: Sci Rep. 2023 Mar 31;13:5309. doi: 10.1038/s41598-023-32010-y (PMC10066291; doi:10.1038/s41598-023-32010-y)
Supplement: Supplementary file 1 — Supplementary Figures. [file 41598_2023_32010_MOESM1_ESM.pdf]

Supporting Information: *Passive atomic-scale optical sensors for mapping light flux in ultra-small cavities*

Pavao Andričević[1*], Elaine L. Sellwood[1], Martha-Cary Eppes[2], Myungho Kook[1], Mayank Jain[1]

[1]Department of Physics, Technical University of Denmark, DTU Risø campus, 4000 Roskilde, Denmark
[2]Department of Geography & Earth Sciences, University of North Carolina at Charlotte, Charlotte, NC, USA

*Corresponding Author: proan@dtu.dk

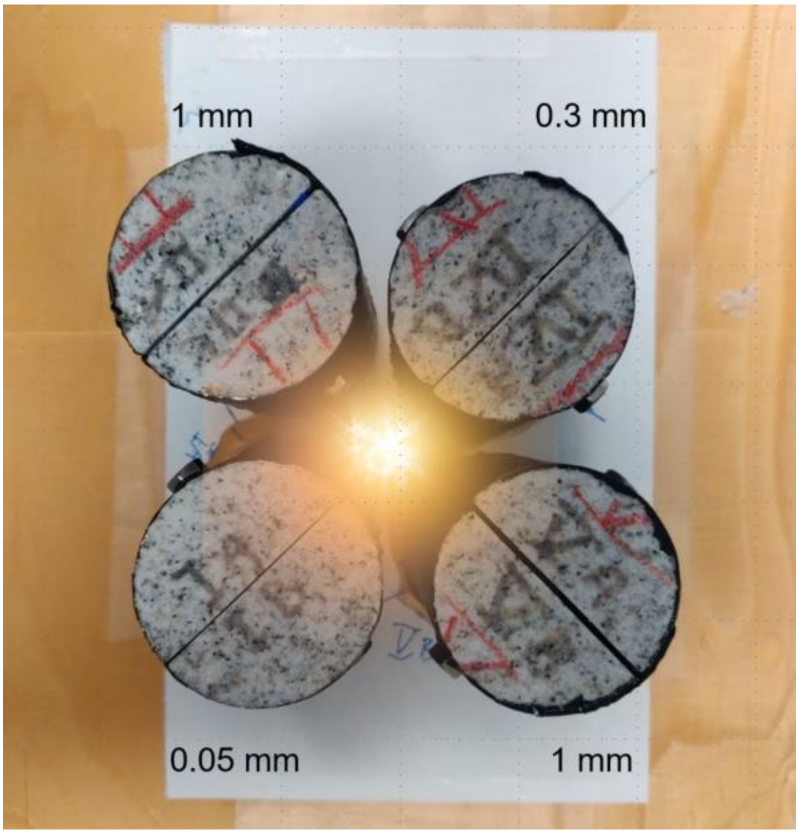

Figure S1.
Optical image of the 4 cores positioned under the solar simulator (light source position indicated).

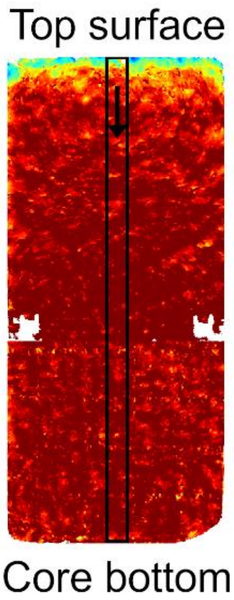

Figure S2.

The black box represents the area from which luminescence values were taken for constructing depth profiles for the time-dependent measurements. A narrow 5 mm segment was chosen, not to be affected by the shading of the isolating tape around the cores as well as the spacers at the edges.

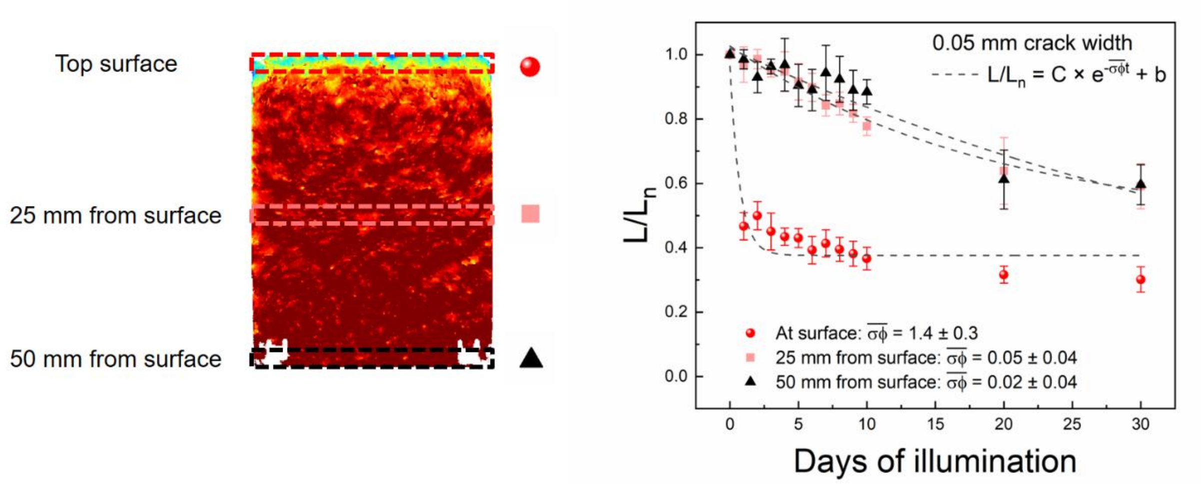

Figure S3.

Average values from three segments at three different depths from the top surface were chosen to represent the dependency of bleaching with time of illumination. Segments are represented as areas bounded by dashed lines, corresponding to the symbol at their immediate right.

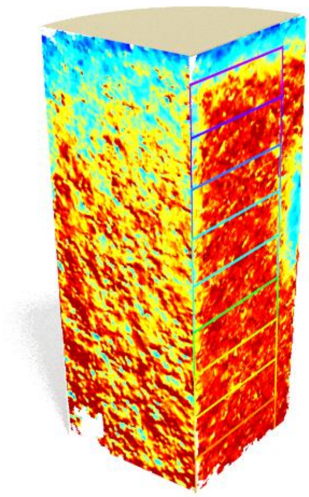

Figure S4.

Boxes of 5 mm width in various colors representing segments at different depths down the crack from which L-X depth profiles were taken. The first 8 mm were disregarded, as bleaching form the top surface would have a significant influence

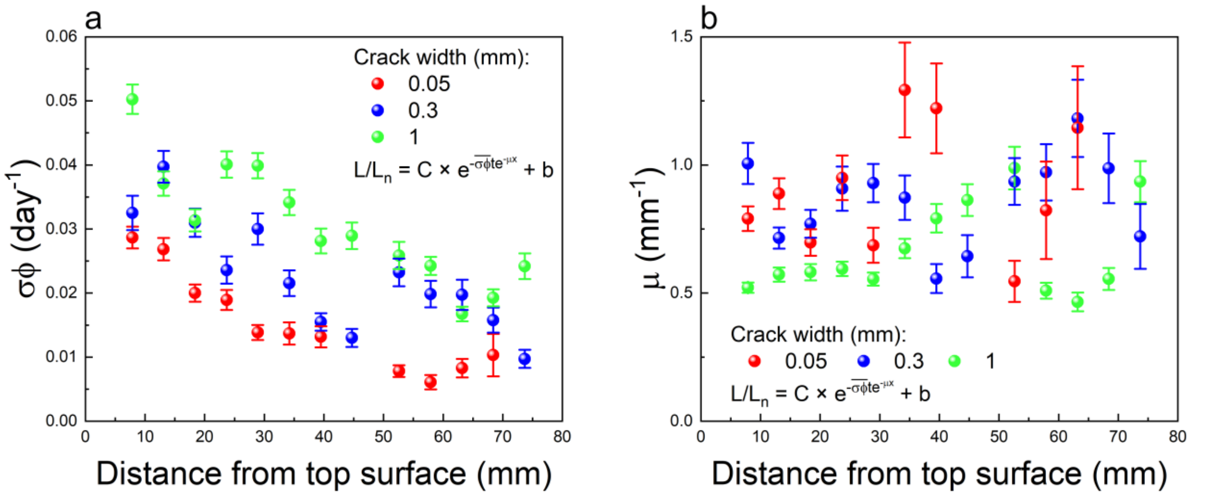

Figure S5.

(a) Decay rates ($\overline{\sigma\varphi_0}$) and (b) attenuation coefficients (μ) obtained from the first order model fitted on the IRPL$_{955}$ L-X depth profiles.

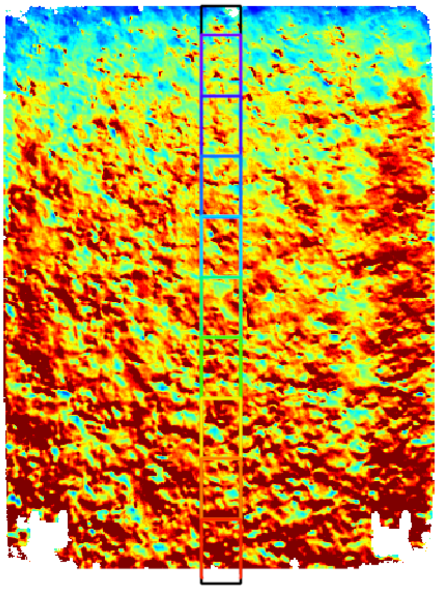

Figure S6.

Illustration of segments down the crack surface from which $IRPL_{955}$ luminescence values were extracted for the time-dependent model.

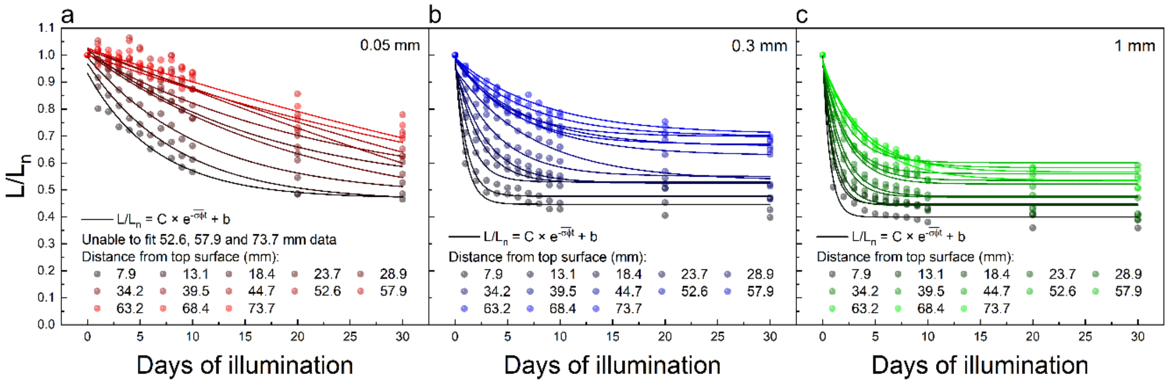

Figure S7.

Time-dependent fits of the $IRPL_{955}$ luminescence values.

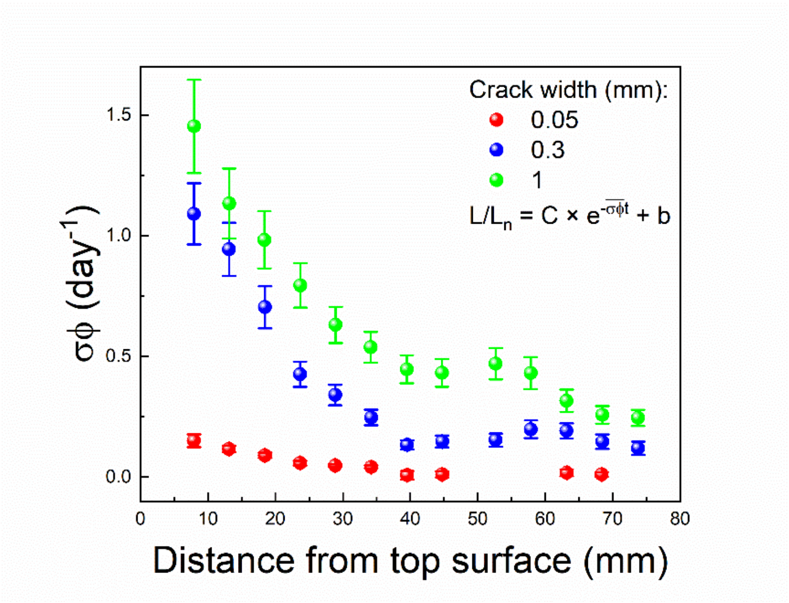

Figure S8.

Decay rates in dependence of distance from the top surface obtained by the time-dependent fits.

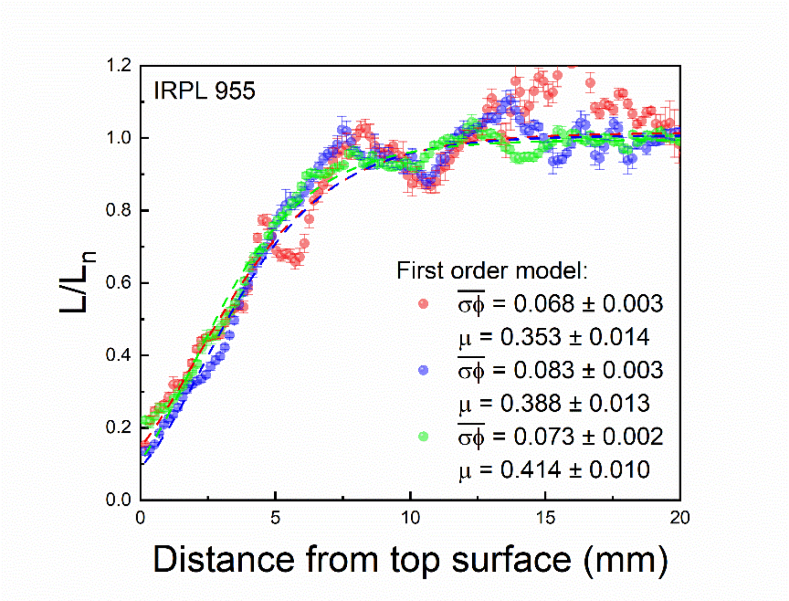

Figure S9.

Depth profiles from the top surface (L-Y) taken from the $IRPL_{955}$ maps of the perpendicular cuts.

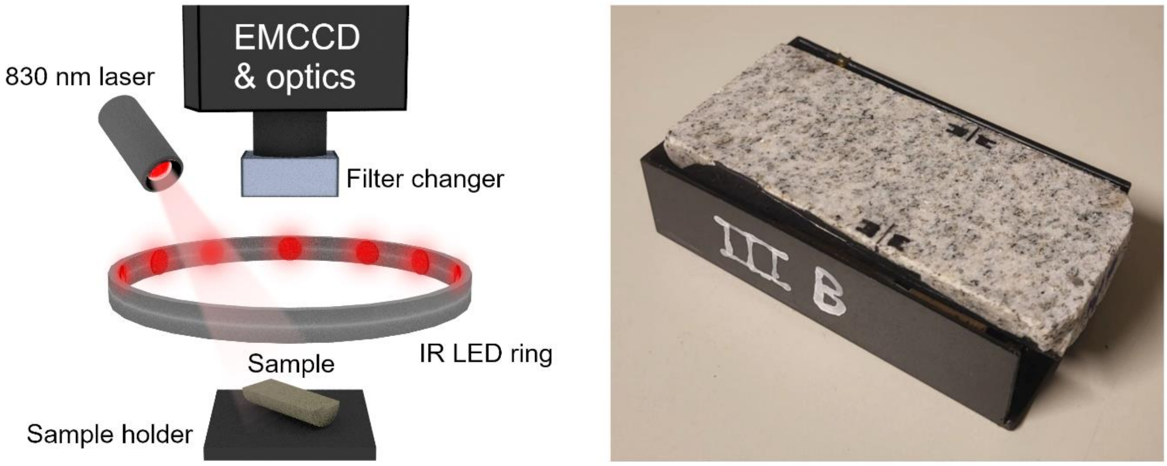

Figure S10.

Schematic of the IRPL setup and image of a granite rock half in a sample holder.
